# Exosomes Released From Human Bone Marrow–Derived Mesenchymal Stem Cell Attenuate Acute Graft-Versus-Host Disease After Allogeneic Hematopoietic Stem Cell Transplantation in Mice

**DOI:** 10.3389/fcell.2021.617589

**Published:** 2021-04-06

**Authors:** Ke-Liang Li, Jin-Yan Li, Gui-Ling Xie, Xiao-Yan Ma

**Affiliations:** Department of Pediatrics, Rizhao People’s Hospital, Rizhao, China

**Keywords:** human bone marrow–derived mesenchymal stem cell–derived exosomes, acute graft-versus-host disease, T-cell subpopulation, inflammatory response, flow cytometry

## Abstract

**Objective:**

Mesenchymal stromal cell–derived exosomes have been applied for the treatment of several immune diseases. This study aimed to explore the effect of human bone marrow–derived mesenchymal stem cell (hBMSC)–derived exosomes on acute graft-versus-host disease (aGVHD) after allogeneic hematopoietic stem cell transplantation (HSCT).

**Methods:**

hBMSC were cultured, and the culture supernatants were then collected to prepare exosomes using total exosome isolation reagent from Invitrogen. Mouse aGVHD model was established by allogeneic cell transplantation and injected with hBMSC-derived exosomes (Msc-exo) via tail vein. Exosomes from human fibroblast (Fib-exo) were used as the treatment control. The effects of Msc-exo on dendritic cells, CD4^+^, and CD8^+^ T cells in aGVHD mice were analyzed through flow cytometry. The impact on inflammatory cytokines was tested by ELISA. Besides, the body weight, survival rate, and clinical score of treated mice were monitored.

**Results:**

Msc-exo were successfully prepared. aGVHD mice injected with Msc-exo led to 7–8-fold increase of the CD8α^+^ conventional dendritic cells (cDCs) and CD11b^+^ cDCs compared with the controls. In addition, Msc-exo altered the T help and Treg subpopulation, and decreased the cytotoxicity and proliferation of cytotoxic T cells to favor inflammatory inhibition in aGVHD mice. Mice that received Msc-exo exhibited decreased weight loss and reduced aGVHD clinical score in a time-dependent manner as well as reduced lethality compared with Fib-exo treated or untreated control. Furthermore, the levels of IL-2, TNF-α, and IFN-γ were decreased, as well as the level of IL-10 was increased after Msc-exo treatment *in vivo* and *in vitro*.

**Conclusion:**

hBMSC-derived exosomes could attenuate aGVHD damage and promote the survival of aGVHD mice by regulating the DC and T-cell subpopulation and function, and lead to inhibited inflammatory response in aGVHD mice.

## Introduction

As the only therapeutic method, allogeneic hematopoietic stem cell transplantation (HSCT) has been widely applied for the treatment of various hematological malignancies (Passweg et al., 2017). Notably, despite the substantial advances in transplant-related technologies such as donor lymphocyte infusion, novel immunosuppressive drugs, and supportive therapy, unsatisfactory prognosis and poor survival remain an issue due to the severe complications after allogeneic HSCT (Sahin et al., 2016). The high mortality after allogeneic HSCT is primarily caused by relapse of disease and also by acute graft-versus-host disease (aGVHD) to some degree (Ferrara et al., 2009; Sahin et al., 2016). Currently, the prevention of aGVHD are mainly achieved by the utilization of a wide variety of drugs such as steroids and calcineurin inhibitors, and the prophylactic effect is poor (Lai et al., 2014). Thus, it is urgent to develop novel effective prevention methods for aGVHD.

Mesenchymal stromal or stem cells (MSCs) are identified as pluripotent cells that present in various tissues, including cord blood, adipose, and bone marrow (Ullah et al., 2015). Due to the unique regenerative and immunoregulatory properties, MSCs have been concerned as therapeutic candidates for several immune diseases such as multiple sclerosis (Connick et al., 2012) and Crohn disease (Dave et al., 2017). Previous clinical studies have suggested that MSC infusion can effectively prevent aGVHD after allogeneic HSCT (Wernicke et al., 2011; Chen et al., 2015; Zhao K. et al., 2015). In addition, MSC infusion can reduce the incidence and severity of chronic GVHD (Zhao K. et al., 2015). However, the prophylactic and therapeutic effects of MSCs are compromised due to uncontrolled differentiation. Besides, only the minority of MSCs could enter the target organs, and the most MSCs remain in the lungs and increase the risk of pneumonia-related death (Forslöw et al., 2012; Haarer et al., 2015). The extent to which MSCs can improve patient outcomes remains unclear because of the significant heterogeneity in the details of disease diagnosis, treatment strategies, and MSC preparation method across clinical studies of MSCs (Rizk et al., 2016).

The immunomodulatory property of MSCs often depends on their secretion, thus, non–cell-based therapy may be an alternative to MSC-based therapy (Salem and Thiemermann, 2010; Andaloussi et al., 2013; Lai et al., 2013). Exosomes are a kind of biological vesicles with a size of 30–120 nm that originated within the endosomal compartment and released from multivesicular bodies (Scott et al., 2014). It has been reported that MSCs can exert immunoregulatory effects through exosomes (Lai et al., 2013). Notably, a recent study has reported an individual treatment for therapy-refractory aGVHD by MSC-derived exosomes, and the improved clinical GVHD symptoms are observed (Kordelas et al., 2014). Therefore, MSC-derived exosomes may be a potential therapeutic method for aGVHD after allogeneic HSCT.

In the present study, human bone marrow–derived mesenchymal stem cell (hBMSC)–derived exosomes were prepared, and the immunoregulatory effects of hBMSC-derived exosomes on aGVHD mice induced by allogeneic HSCT were explored.

## Materials and Methods

### Preparation of Exosomes

hBMSCs at the third passage were purchased from Shanghai Obio Technology (China). Human dermal fibroblasts purchased from the same company were used as control. Cells were maintained in Dulbecco’s modified Eagle’s medium (DMEM; Gibco, Carlsbad, CA, United States) containing 10% exosome-depleted fetal bovine serum (Gibco) with standard incubation conditions (5% CO_2_ and 37°C). Exosomes were extracted using total exosome isolation reagent (Invitrogen, Gaithersburg, MD, United States). Briefly, the culture medium of hBMSCs at the fourth to sixth passage were collected and centrifuged at 2,000 × *g* for 30 min at 4°C. Then, the supernatant was mixed with isolation agent and incubated overnight at 4°C. Followed by the centrifugation of the mixture at 10,000 × *g* at 4°C for 1 h, the supernatant was removed and hBMSC-derived exosomes were obtained. Lastly, hBMSC-derived exosomes were resuspended in 100 μl of pre-cooled PBS and stored at −20°C.

### Characterization of hBMSC-Derived Exosomes

The characterization of hBMSC-derived exosomes was observed using transmission electron microscopy (TEM; HT7700; Hitachi, Japan). Briefly, hBMSC-derived exosomes were fixed in 2% paraformaldehyde. Followed by negative staining with 3% phosphotungstic acid for 5 min, hBMSC-derived exosomes were visualized under TEM. Dynamic light scattering (DLS) was performed to measure the particle size of hBMSC-derived exosomes by Malvern laser particle size analyzer (Malvern, United Kingdom).

### Identification of hBMSC-Derived Exosomes

The markers of exosomes, including CD9, CD63, and CD81, were detected by western blot. In brief, hBMSC-derived exosomes were quantitated by bicinchoninic acid kit (Beyotime, Shanghai, China). Following sample separation and transfer onto PVDF membranes, membranes were immerged in 5% non-fat milk for 1 h. Next, primary antibodies of CD9, CD63, and CD81 (1:800; Sigma), respectively, were used for immunoblotting of the membranes overnight at 4°C. Then, membranes were reacted with secondary antibody (1:1,000; Beyotime) for 2 h at room temperature. The signals were revealed using enhanced chemiluminescence Plus reagent (Beyotime).

### Incubation of hBMSC-Derived Exosomes With Immune Cells

Human peripheral blood mononuclear cells (hPBMCs) were purchased from Shanghai Obio Technology (China) and maintained in DMEM (Gibco) containing 10% fetal bovine serum (Gibco) with standard incubation conditions (5% CO_2_ and 37°C). hPBMCs were exposed to 5 μg/ml of phytohemagglutinin (PHA) and 5 ng/ml of recombinant human interleukin 2 (rhIL-2), and then incubated with 100 μg/ml Msc-exo or Fib-exo (control). After cultured for 6 days, the cell culture supernatant was collected for the following ELISAs.

### Mouse aGVHD Model and Treatment

The animal study was reviewed and approved by Ethics Committee of Rizhao People’s Hospital. Healthy male C57BL/6 and BALB/c mice (6–8 weeks old and weighing 120–150 g, purchased from Charles River, Beijing, China) were used for the following experiments after 1 week of acclimation. The mouse model of aGVHD was established by allogeneic cell transplantation as previously described (Lai et al., 2018). C57BL/6 mice were sacrificed by cervical dislocation method. The femur and tibia were taken and cut both sides, then the medullary cavity was repeatedly washed with PBS. The bone marrow cell suspension was filtered through a 40-μm filter and then centrifuged at 1,000 rpm for 5 min. Red blood cell lysate was added to remove blood cells. After centrifugation, bone marrow cells were collected, and the final concentration was adjusted to 6 × 10^7^/ml. Meanwhile, spleen was obtained to collect spleen cells, similar to the method of bone marrow cells, and the final concentration of spleen cells was adjusted to 3 × 10^7^/ml. On day 0, recipient BALB/c mice received total body irradiation with 8 Gy per mouse at a dose rate of 0.5 Gy/min. Four hours post-irradiation, mice were injected with 100 μl bone marrow cells and 100 μl spleen cells via tail vein. Mice in the exosomes injection group (*n* = 10) were injected with 200 μg exosomes per mouse via tail vein on the day of transplantation. After the treatment, the body weight and survival situation of mice were monitored every 3 days to plot body weight curve and survival curve, until death of animal or termination of the study on day 30. Meanwhile, five indicators, including body weight, activity, posture, skin texture, and hair removal, were scored as 0–2 based on severity, and the total scores were considered as the clinical score. The animals in different treatment groups were not randomly housed. The clinical scores of aGVHD mice were evaluated by two independent researchers in a blind way.

Recombinant mouse IL-10 was purchase from GeneScript and was intravenously injected into aGVHD mice with a dose of 5 μg/kg every other day (injection volume 50 μl) in the IL-10 treatment group starting from day 0. For TNF-α inhibition, mice were injected intraperitoneally with etanercept (Merck) at a dose of 5 mg/kg body weight every 3 days starting from day 0 and the animals in the control group received injections of saline.

### Flow Cytometry

Mononuclear cells were immunostained with various combinations of fluorescent antibodies against CD3, CD4, CD8, IL-17, Foxp3, Ki-67, CD11b, CD8α, and granzyme B (eBioscience, San Diego, CA, United States). For intracellular cytokine detection, the cells were restimulated with PMA and ionomycin for 4 h in the presence of Golgi-stop. Labeled cells were enumerated by Canto II flow cytometer (BD Biosciences, San Jose, CA, United States). Data were analyzed by BD FACSDiva software 6.0 (BD).

### ELISA

The culture supernatant as well as serum samples on day 5 and day 10 after transplantation were measured by commercial ELISA kits (Boster, Wuhan, China) for the concentrations of IL-2, IL-10, INF-γ, and TNF-α according to the manufacturer’s instructions. Briefly, supernatant samples (100 μl) were incubated in a 96-well plate which coated with antigen in each well at 37°C for 1.5 h, followed by incubation with biotinylated antibody (100 μl) at 37°C for 1 h. Afterward, each well was added with avidin peroxidase (100 μl) at 37°C for 30 min, substrate solution (90 μl) in the dark at 37°C for 15 min, and stopping solution (50 μl) in turn. Lastly, absorbance at 450 nm was detected by a microplate reader (Thermo, Philadelphia, PA, United States) to calculate the level of each cytokine.

### Statistical Analysis

SPSS Statistics 20.0 software (IBM, Armonk, NY, United States) was used for data statistical analysis. Data were expressed as the mean ± SD. For a two-group comparison, unpaired two-tailed Student’s *t*-test was used. The differences among multiple groups were analyzed by one-way ANOVA followed by multiple comparison with Tukey test. Survival curves were estimated by the Kaplan–Meier method, and the statistical differences were estimated by the log-rank test. *P* value <0.05 indicated statistical significance.

## Results

### Characteristics of hBMSC-Derived Exosomes

**FIGURE 1:**
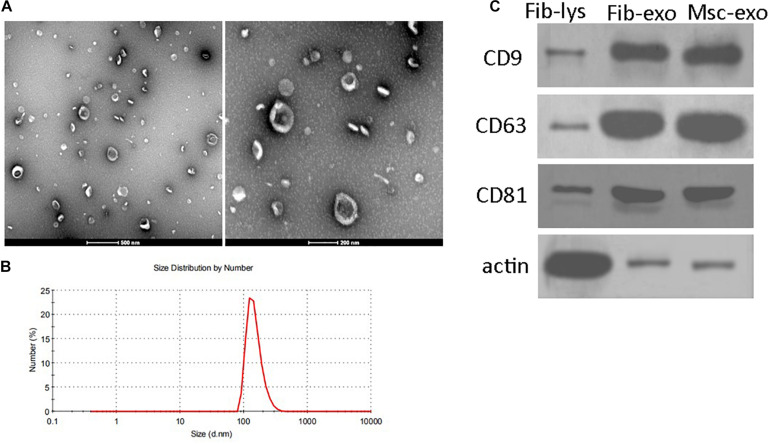
Characteristics of human bone marrow–derived mesenchymal stem cell (hBMSC)–derived exosomes. **(A)** Representative images of hBMSC-derived exosomes under transmission electron microscopy. Scale bar: 500 and 200 nm. **(B)** Size distributions of hBMSC-derived exosomes determined by dynamic light scattering. **(C)** Positive expression of CD9, CD63, and CD81 in isolated exosomes by western blot. β-Actin was used as the control for western blot. Fib-lys, the total cell lysate of human fibroblasts; Fib-exo, the exosomes from human fibroblasts; Msc-exo, the exosomes from human bone marrow–derived mesenchymal stem cells.

hBMSC-derived exosomes were prepared through sequential total exosome isolation reagent from Invitrogen. Exosomes isolated from human fibroblast cells using the same method were used as the treatment control. TEM image presented a group of heterogeneous spheroids with the size of approximately 100 nm, which was the typical shape of exosomes (Figure 1A). Similarly, DLS also showed that the hydrodynamic diameter of hBMSC-derived exosomes was observed to be around 100 nm (Figure 1B). In addition, western blotting results showed the enrichment of the markers of exosomes such as CD9, CD63, and CD81 in isolated samples (Figure 1C). These results confirmed the successful preparation of hBMSC-derived exosomes.

### Effect of hBMSC-Derived Exosomes on the DC Subpopulation in aGVHD Mice

**FIGURE 2:**
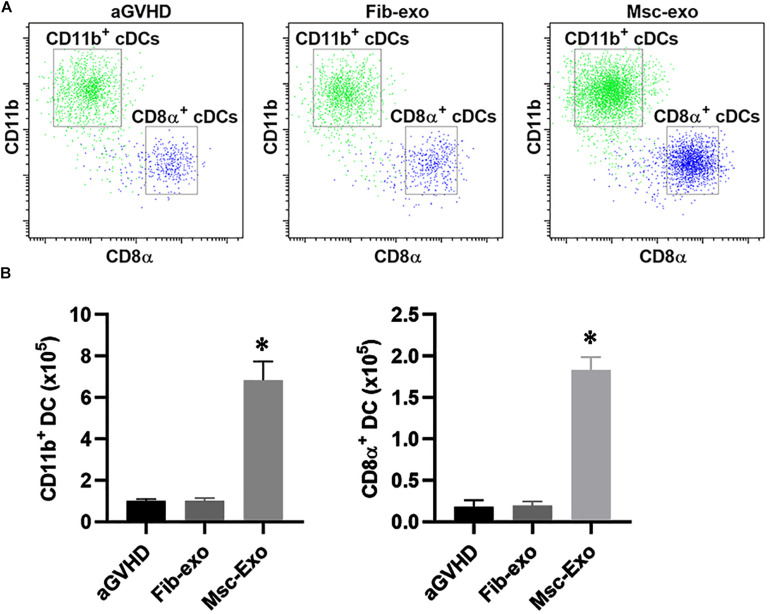
Effect of Msc-exo on the dendritic cell (DC) subpopulation in acute graft-versus-host disease (aGVHD) mice. aGVHD mice were injected with either Fib-exo or Msc-exo via tail vein, and the number of CD11b^+^ and CD8α^+^ conventional dendritic cells (cDCs) in the spleen was analyzed by flow cytometry 10 days after exosome injection. **(A)** Representative plots showing the ratio of CD11b^+^ and CD8α^+^ cDCs. **(B)** CD11b^+^ and CD8α^+^ cDCs in the spleen were enumerated 10 days after exosome injection. Data were representative of three independent experiments where *n* = 5 in each group. ^∗^*p* < 0.05.

The mouse model of aGVHD were established by allogeneic cell transplantation, and then injected with hBMSC-derived exosomes (Msc-exo) or human fibroblast–derived exosomes (Fib-exo) as the treatment control via tail vein. The beneficial effects of hBMSC-derived exosomes on immune cells in aGVHD mice were investigated. Dendritic cells (DCs) are the most potent antigen-presenting cells and are important regulators of GVHD (Toubai et al., 2014). Previous studies have shown that host-derived CD8α^+^ DCs and CD11b^+^ DCs reduced acute GVHD by regulating IL-10 expression and inducing Treg and T helper in mice (Yamazaki et al., 2008; Toubai et al., 2010). Therefore, we analyzed the number of CD8α^+^ DCs and CD11b^+^ DCs in mice spleen 10 days after exosomes treatment by flow cytometry (Figures 2A,B). We found hBMSC-derived exosomes (Msc-exo) led to 7–8-fold increase of the CD8α^+^ cDCs and CD11b^+^ cDCs compared with human fibroblast–derived exosome treated (Fib-exo) or the untreated mice. These data suggested the beneficial effects of Msc-exo on aGVHD.

### Effect of hBMSC-Derived Exosomes on the T-Cell Subpopulation in aGVHD Mice

**FIGURE 3:**
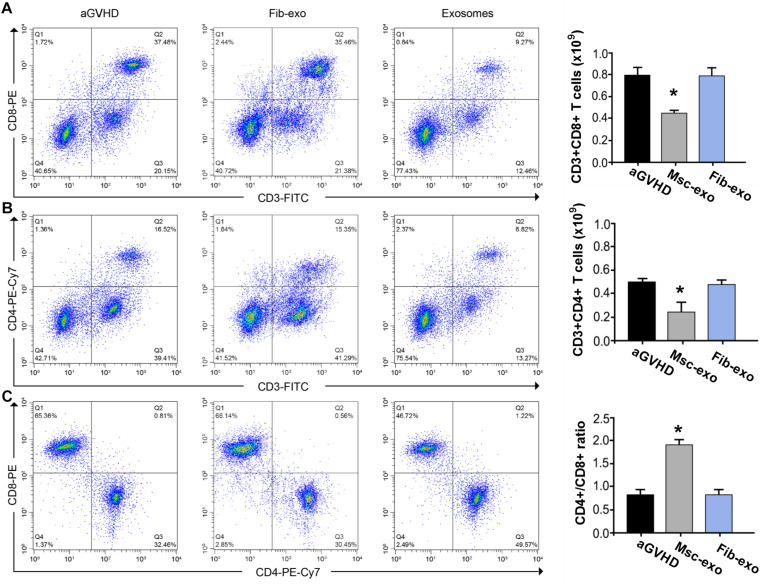
Human bone marrow–derived mesenchymal stem cell (hBMSC)–derived exosomes altered T-cell subpopulation in acute graft-versus-host disease. **(A)** The number of CD3^+^CD8^+^ T cells of blood samples in the control and Msc-exo treated mice. **(B)** The number of CD3^+^CD4^+^ T cells of blood samples in the control and Msc-exo treated mice. **(C)** The ratio of CD4^+^ to CD8^+^ T cells in the control and Msc-exo treated mice. *p < 0.05. Data were representative of two to three independent experiments where *n* = 5 in each group.

**FIGURE 4:**
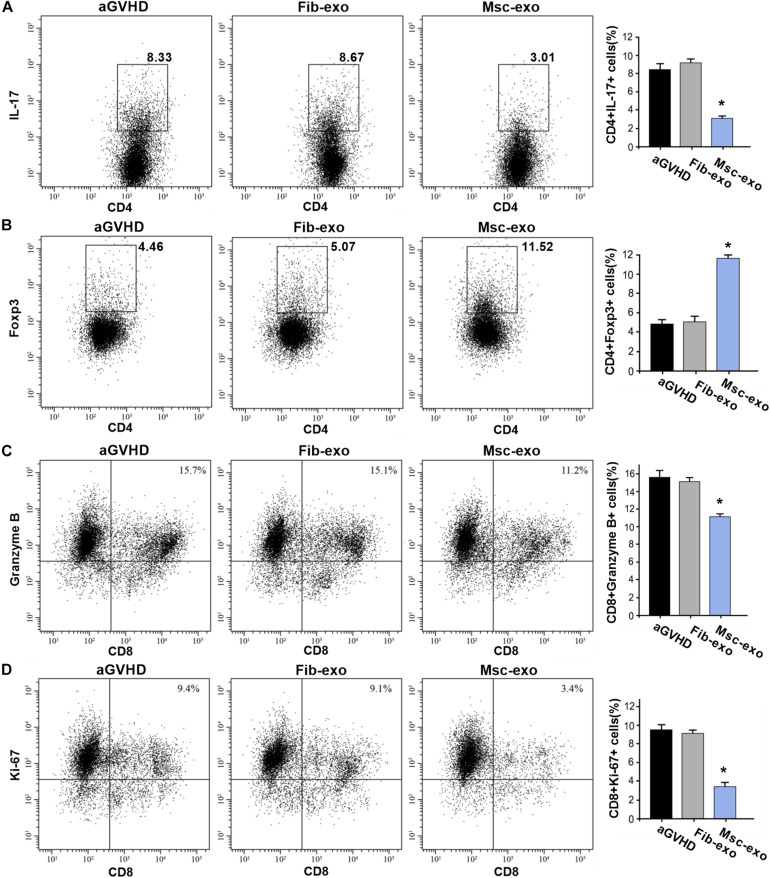
Msc-exo could regulate the function and biology of T-cell subpopulation in acute graft-versus-host disease (aGVHD) mice. aGVHD mice were injected with either Fib-exo or Msc-exo, or untreated. 10 days after injection, Th17 (IL-17^+^CD4^+^) and Treg (Foxp3^+^CD4^+^) in the blood were analyzed by flow cytometry. The proliferation and cytotoxicity of T cells were analyzed with markers of granzyme B and Ki-67 using flow cytometry. **(A)** Representative plots (left) and the quantified data of IL-17^+^CD4^+^ T cells in the exosome treated and untreated mice. **(B)** Foxp3^+^CD4^+^ T cells in the treated and untreated mice. **(C)** The cytotoxicity of T cells was measured by the expression of Granzyme B through flow cytometry. **(D)** The proliferation of T cells in the exosome treated and untreated aGVHD mice. *N* = 3–4 in each group. Data were representative of at least two independent experiments. *p < 0.05.

As DCs are critical for priming T-cell responses, we next analyzed T-cell subpopulation in hBMSC-derived exosome treated aGVHD mice. Flow cytometry assay revealed that hBMSC-derived exosomes remarkably inhibited the number of CD3^+^CD8^+^ T cells in the blood on day 10 after transplantation (*p* < 0.05, Figure 3A). Meanwhile, a reduction of CD3^+^CD4^+^ T cells was found in the blood of aGVHD mice that received Msc-exo compared with Fib-exo control mice (*p* < 0.05, Figure 3B). Notably, hBMSC-derived exosomes conspicuously increased the ratio of CD4^+^ to CD8^+^ T cells on day 10 after transplantation (*p* < 0.05, Figure 3C). Moreover, the relative balance of Th17 and regulatory T cells (Tregs) is considered as a critical indicator to induce pro- or anti-inflammatory reactions and maintain immune homeostasis (Lee et al., 2009). By flow cytometry, we found that Msc-exo injection in aGVHD mice decreased the blood CD4^+^IL-17^+^ T helper cells while increased the CD4^+^Foxp3^+^ Tregs compared with the Fib-exo group or untreated mice on day 10 after transplantation (Figures 4A,B). In addition to the activation of T-cell subpopulation, we analyzed the effect of Msc-exo on CD8^+^ T-cell cytotoxicity and proliferation through monitoring the expression of marker protein granzyme B and Ki-67 using flow cytometry on day 10 after transplantation (Figures 4C,D). We found Msc-exo injection resulted in decreased number of granzyme B or Ki-67 positive CD8^+^ T cells in aGVHD mice compared with Fib-exo treated or untreated mice. Overall, these data suggested that Msc-exo could regulate the function and biology of T-cell subpopulation in aGVHD mice.

### Effect of hBMSC-Derived Exosomes on the Release of Inflammatory Cytokines *in vitro* and *in vivo*

**FIGURE 5:**
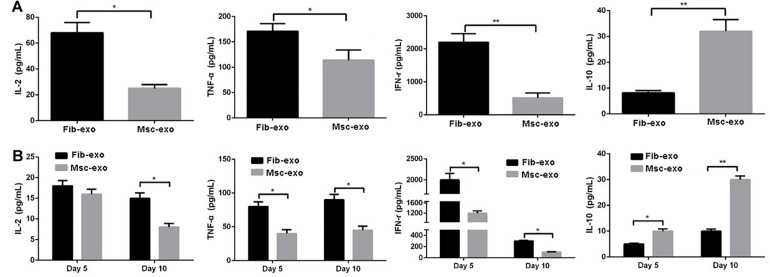
Human bone marrow–derived mesenchymal stem cell (hBMSC)–derived exosomes inhibited inflammatory response related to acute graft-versus-host disease (aGVHD) *in vitro* and *in vivo*. **(A)** Levels of inflammatory cytokines in culture supernatants produced by PHA/rhIL-2-activated human peripheral blood mononuclear cells with or without hBMSC-derived exosomes. **(B)** Levels of inflammatory cytokines in serum samples in aGVHD mice that did or did not receive hBMSC-derived exosomes on day 5 and day 10 after transplantation. *p < 0.05, **p < 0.01. IL-2, interleukin-2; INF-γ, interferon-γ; TNF-α, tumor necrosis factor-α. *N* = 3–8 in each group. Data were representative of at least two independent experiments.

Furthermore, inflammatory cytokines such as TNF-α, IFN-γ, IL-2, and IL-10 were measured to evaluate the role of hBMSC-derived exosomes on inflammatory response related to aGVHD. PHA and rhIL-2 were used to activate hPBMCs *in vitro*, and the results showed that the contents of pro-inflammatory cytokines, including IL-2, TNF-α, and IFN-γ, were significantly decreased, whereas the content of anti-inflammatory cytokine IL-10 was obviously increased after hBMSC-derived exosome treatment in activated hPBMCs (*p* < 0.05, Figure 5A). Accordingly, *in vivo* experiments revealed that hBMSC-derived exosomes prominently inhibited the contents of IL-2, TNF-α, and IFN-γ, while they remarkably elevated IL-10 level (*p* < 0.05, Figure 5B) on day 5 and day 10 after transplantation.

### Effect of hBMSC-Derived Exosomes on the Manifestations of aGVHD Mice

**FIGURE 6:**
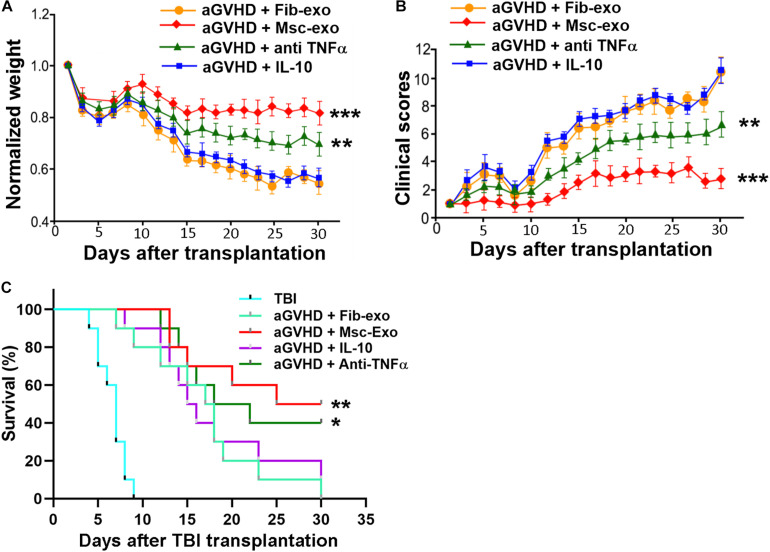
Human bone marrow–derived mesenchymal stem cell (hBMSC)–derived exosomes alleviated the manifestations of acute graft-versus-host disease (aGVHD) in mice. Mice in the exosome injection group were injected with 200 μg exosomes per mouse via tail vein on the day of transplantation. Recombinant mouse IL-10 was intravenously injected into aGVHD mice with a dose of 5 μg/kg every other day (injection volume 50 μl) in the IL-10 treatment group starting from day 0. For TNF-α inhibition, mice were injected intraperitoneally with etanercept (Merck) at a dose of 5 mg/kg body weight every 3 days starting from day 0 and the animals in the control group received injections of saline. No exosome was used in IL-10 and etanercept treated mice. **(A)** Weight changes of aGVHD mice in each treatment group. **(B)** Clinical score of aGVHD mice in each treatment group. **(C)** Survival curves of aGVHD mice in each treatment group. TBI, total body irradiation. ***p < 0.001, **p < 0.001, *p < 0.05. *N* = 10 in each group.

Since Msc-exo showed beneficial immune modulation effects in aGVHD mice, we tested whether Msc-exo could promote the survival of aGVHD mice. hBMSC-derived exosomes were used to treat aGVHD mice and compared with the Fib-exo treated control mice. We found that mice received hBMSC-derived exosomes exhibited increased weight and reduced aGVHD clinical score in a time-dependent manner compared with Fib-exo treated aGVHD mice (*p* < 0.001, Figures 6A,B). In addition, hBMSC-derived exosomes obviously reduced the lethality related to aGVHD (*p* < 0.001, Figure 6C). To further test whether the effects of Msc-exo was mediated by cytokines such as IL-10 and TNF-α, aGVHD mice were administrated with IL-10 intravenously or anti-TNF-α drug etanercept intraperitoneally, respectively (Figures 6A–C). No exosome was used in IL-10 and etanercept treated mice. We found IL-10 had no effects on the body weight, clinical scores, and survival rate of aGVHD mice. In contrast, block the binding of TNF-α to its receptor using etanercept showed moderate beneficial effects in aGVHD mice, albeit to a lesser extent than Msc-exo treatment. Overall, these data indicated that Msc-exo could improve the survival of aGVHD mice, and the effect was likely mediated in part by inhibiting the secretion of pro-inflammatory cytokine TNF-α.

## Discussion

Extensive clinical trials have evaluated the therapeutic effects of MSCs as a cellular therapy (Mazzini et al., 2010; Squillaro et al., 2016; Turinetto et al., 2016). Importantly, increasing evidences have shown that MSCs exert the immunomodulatory functions largely by secreting various soluble factors and cytokines (Spees et al., 2016). Exosomes as the secretions from MSCs contain various proteins, lipids, DNA, mRNA, and miRNA, which can mediate cell-to-cell communication (Scott et al., 2014). Accumulated studies have shown that MSC-derived exosomes play an important role in many diseases, such as myocardial injury (Zhao Y. et al., 2015), nerve tissue regeneration (Mao et al., 2019), and lung injury (Monsel et al., 2016). In particular, the immunosuppressive effects of MSC-derived exosomes on GVHD have been widely reported. For example, Fujii et al. have found that MSC-derived exosomes alleviated GVHD and prolonged the overall survival of allogeneic mouse GVHD model (Fujii et al., 2018). A similar therapeutic effect of MSC-derived exosomes was reported by Zhang et al. in xenogeneic GVHD mouse model (Zhang et al., 2018). In addition, a recent clinical study has preliminarily confirmed the ameliorated effect of MSC-derived exosomes on GVHD damage in a patient with resistant grade IV aGVHD (Kordelas et al., 2014). In this study, we found that hBMSC-derived exosomes altered DC and T-cell subpopulation, and inhibited inflammatory response in aGVHD mice, resulting in reduced aGVHD clinical score and increased survival rate of aGVHD mice. These results suggested the potential of hBMSC-derived exosomes as a convenient and safe non–cell-based therapy.

Innate immune cells such as monocytes and macrophages could be activated by pathogen-associated molecular pattern molecules, thereby stimulating inflammatory cells and increase the levels of pro-inflammatory cytokines (Liu and Cao, 2016). aGVHD remains a lethal complication in hematopoietic cell transplantation patients even in HLA-matched donor–recipient pairs (Hülsdünker and Zeiser, 2015). Although the pathogenesis of aGVHD has not been fully understood, the pivotal regulating roles of activated T lymphocyte have been acknowledged in the occurrence of GVHD (Hülsdünker and Zeiser, 2015). The relative balance of Th17 and Treg cells is considered as a critical indicator to induce pro- or anti-inflammatory reactions and maintain immune homeostasis (Lee et al., 2009). A previous study has demonstrated that MSC-exosomes exhibited immunosuppressive effects by inhibiting T-cell activation in autoimmune diseases (Bai et al., 2017). A recent study also has shown that the improved survival and ameliorated pathologic damage induced by MSC-exosomes are closely associated with increased Treg cells and reduced Th17 cells in chronic GVHD (Lai et al., 2018). In this study, we found Msc-exo induced similar changes in Th17 and Treg cells in our acute GVHD mice model. Our data all showed that hBMSC-derived exosomes significantly decreased the number of CD8^+^ cytotoxic T cells as well as inhibited the number of CD3^+^CD4^+^ T cells in aGVHD mice. Since the activation of CD4^+^ T helper cells and CD8^+^ cytotoxic T cells can promote inflammation response by secreting pro-inflammatory cytokines, such as IL-2, TNF-α, and IFN-γ (Smith et al., 2004), the reduction of the ratio is an important indicator of the decreased immune function (Smith et al., 2004). Consistently, this study revealed that hBMSC-derived exosomes inhibited the levels of IL-2, TNF-α, and IFN-γ as well as increased the level of IL-10, which further suggested that hBMSC-derived exosomes showed an anti-inflammatory effect by regulating the changes of T-cell subpopulation. Interestingly, we found that systemic administration of anti-TNF-α drug showed moderate beneficial effects in aGVHD mice, whereas IL-10 had no obvious effects, indicating that the beneficial effect was likely mediated in part by inhibiting the secretion of pro-inflammatory cytokine TNF-α.

Although promising effects of MSCs-exo were observed, our study has certain limitations due to the experimental design. Exosomes were given at the time of transplant in all animals rather than at the onset of aGVHD manifestations, which may be barriers to clinical translation. Lack of randomization in mice housing may lead to potential sources of bias. Moreover, the contents of the Msc-exo were not explored. Future studies on the characterization of the contents of Msc-exo are encouraged to elucidate the therapeutic effects in a more precise manner.

## Conclusion

In conclusion, the findings of this study demonstrated that hBMSC-derived exosomes can attenuate aGVHD damage and promote the survival rate of aGVHD mice by regulating the proportion of T-cell subpopulation and inhibiting inflammatory response in aGVHD mice, which provided the potential of hBMSC-derived exosomes as a convenient and safe non–cell-based therapy.

## Data Availability Statement

The original contributions presented in the study are included in the article/supplementary material, further inquiries can be directed to the corresponding author/s.

## Ethics Statement

The animal study was reviewed and approved by the Ethics Committee of Rizhao People’s Hospial.

## Author Contributions

K-LL performed the majority of experiments and analyzed the data. J-YL performed the molecular investigations. G-LX designed and coordinated the research. X-YM wrote the manuscript. All authors contributed to the article and approved the submitted version.

## Conflict of Interest

The authors declare that the research was conducted in the absence of any commercial or financial relationships that could be construed as a potential conflict of interest.
